# Sensor-Based Balance Training with Exergaming Feedback in Subjects with Chronic Stroke: A Pilot Randomized Controlled Trial

**DOI:** 10.3390/brainsci14090917

**Published:** 2024-09-13

**Authors:** Alex Martino Cinnera, Irene Ciancarelli, Serena Marrano, Massimiliano Palagiano, Elisa Federici, Alessio Bisirri, Marco Iosa, Stefano Paolucci, Giacomo Koch, Giovanni Morone

**Affiliations:** 1 Scientific Institute for Research, Hospitalization and Health Care IRCCS Santa Lucia Foundation, 00142 Rome, Italy; sere.marrano@gmail.com (S.M.); palagiano.massimiliano@gmail.com (M.P.); s.paolucci@hsantalucia.it (S.P.); 2 Department of Life, Health and Environmental Sciences, University of L’Aquila, 67100 L’Aquila, Italy; irene.ciancarelli@univaq.it (I.C.); giovanni.morone@univaq.it (G.M.); 3 School of Physiotherapy, Faculty of Medicine and Surgery, University of Rome Tor Vergata, 00133 Rome, Italy; federicielisa@gmail.com; 4 Villa Sandra Institute, 00148 Rome, Italy; alessiobisirri@gmail.com; 5 Department of Psychology, Sapienza University of Rome, 00185 Rome, Italy; marco.iosa@uniroma1.it; 6 Department of Neuroscience and Rehabilitation, University of Ferrara, 44121 Ferrara, Italy; giacomo.koch@unife.it; 7 San Raffaele Institute of Sulmona, 67039 Sulmona, Italy

**Keywords:** IMU, rehabilitation, inertial measurement units, force platform, biofeedback

## Abstract

Background: As one of the leading causes of disability in the world, stroke can determine a reduction of balance performance with a negative impact on daily activity and social life. In this study, we aimed to evaluate the effects of sensor-based balance training with exergaming feedback on balance skills in chronic stroke patients. Methods: 21 individuals (11F, 57.14 ± 13.82 years) with a single event of ischemic stroke were randomly assigned to the sensor-based balance training group (SB-group) or the usual care balance training group (UC-group). Both groups received 10 add-on sessions with exergaming feedback (SB-group) or conventional training (UC-group). Clinical and instrumental evaluation was performed before (t0), after (t1), and after one month (t2) from intervention. Participation level was assessed using the Pittsburgh Rehabilitation Participation Scale at the end of each session. Results: The SB-group showed an improvement in postural stability (*p* = 0.02) when compared to the UC-group. In the evaluation of motivational level, the score was statistically higher in the SB-group with respect to the UC-group (*p* < 0.01). Conclusion: Except for the improvement in postural stability, no difference was recorded in clinical score, suggesting a comparable gain in both groups. However, patients undergoing sensor-based training exhibited a higher participation score, ultimately indicating the use of this training to improve the adherence to rehabilitation settings, especially in patients with lower compliance.

## 1. Introduction

Stroke is one of the leading causes of disability worldwide [1], and patients often experience balance impairment, which ultimately impacts the quality of their life [2]. In fact, despite numerous individuals displaying a recovery in their ambulatory capabilities, residual balance challenges often continue to affect those patients during the long-term phase [3]. Both static and dynamic balance disorders are key contributors to an increased risk of falls [4], affecting daily functional performance [5]. While there is empirical support for the effectiveness of balance-focused rehabilitation in stroke survivors, current knowledge is not completely exhaustive. Stroke survivors generally benefit from traditional approaches aiming to restore balance; nevertheless, the efficacy of these methods is not supported by robust scientific validation [6]. This gap in conventional rehabilitative approaches may originate from insufficient direct engagement of the central nervous system during physical therapy exercises. In fact, the presence of inadequate feedback, which is essential for the instantaneous recognition of performance and consequently for the plasticity-dependent learning mechanisms, might not guarantee a proper top-down neural activation [7]. Specifically, integrating appropriate feedback in technologically advanced devices for rehabilitation through audiovisual feedback [8] can promote a cortical activation of specific cerebral areas involved in visual and auditory perception, sensory integration, recognition of movement, re-mapping on the somatosensory and motor cortex, storage in memory, and response control [9,10]. Such audiovisual feedback bolsters patient awareness of their progress and engagement; both conditions encourage participation in task-specific exercises, thus enhancing adherence to the rehabilitation setting [11]. This is achieved by merging multisensory stimuli—visual, auditory, tactile, and somatosensory—produced by systems for immersive virtual feedback [12]. The effectiveness of biofeedback can be further improved when provided as exergaming. Within the neurological rehabilitation context, exergaming exhibited a positive influence on functional recovery [13] and motivational dynamics [14]. A recent comprehensive analysis moderately supports the effectiveness of visual feedback in enhancing balance in chronic stroke survivors, although the efficacy on patients with early and mid-stage stroke is less pronounced [15]. Given the increasing incidence of stroke and its consequent challenges each year [16], there is a pressing need to explore intervention strategies that leverage recent technological advancements for improved balance recovery. In this way, in our previous study, we observed positive results in balance function in patients with subacute stroke who underwent ten sessions of training using an integrated biofeedback system composed by five inertial measurement units (IMUs) and a sensorized force platform. The present study aims to evaluate the effects of sensor-based training with exergaming feedback, performed with an integrated IMUs system and a force platform, on balance functions in individuals with chronic stroke. The results can provide new insight and future directions about the effects of sensor-based balance training in the chronic phase of stroke.

## 2. Materials and Methods

To investigate the effects of sensor-based balance training on balance functions in chronic stroke patients, a randomized two-arm clinical trial was designed. We enrolled adult patients who suffered from a stroke (complete inclusion and exclusion criteria were reported in Table 1).

The study was approved by the local ethics committee, and each participant signed a written informed consent before starting the experimental procedures. All patients were randomly assigned to one of the two study groups using a computer random generation list. Patients included in the sensor-based balance training group (SB-group) underwent 10 sessions of real-time biofeedback therapy performed with an adaptive integrated system composed by five IMUs, a force platform. Patients assigned to the usual-care balance training group (UC-group) performed 10 sessions of conventional exercises for balance rehabilitation with the same duration and intensity as the experimental group.

Each treatment, for both groups, lasted 30 min, and the 10 sessions were distributed over four weeks, with a frequency of 2/3 treatments per week.

### 2.1. Sensor-Based Balance Training

**Figure 1:**
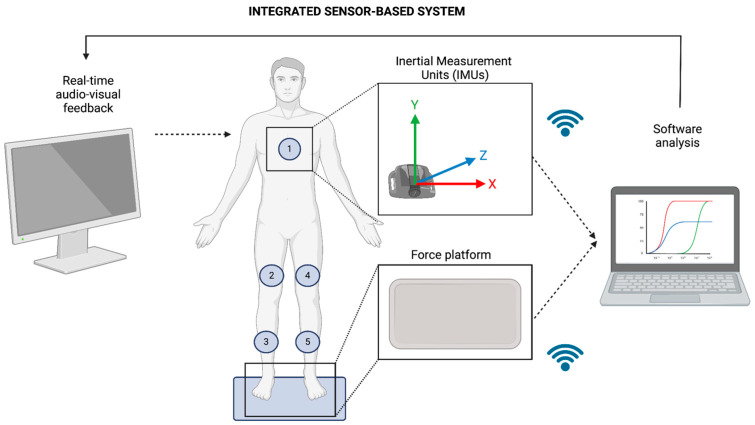
Schematic representation of the sensor-based system used to perform the balance training. The system was composed of five magneto-inertial sensors (1–5) and a force platform connected via Bluetooth to a notebook. The output of feedback, in for of exergaming, was provided on a 32-inch screen. The figure was created with BioRender.com.

For the sensor-based balance training, we used an adaptive integrated system composed of five IMUs and a force platform wirelessly connected to a notebook (RIABLO™—CoRehab, Trento, Italy: v2.0). The system provides real-time audio–visual feedback on movement and balance performance through a screen in the form of exergaming. The five IMUs were attached to the patient through elastic bands and used a wireless Bluetooth connection working at the sampling frequency of 50 Hz [19]. The IMUs were placed 2 mid-thighs and 2 mid-tibial levels on both sides, and the last one in the center of the chest (on the mammillary line) (Figure 1). The training protocol consisted of five exercises: (i) latero-lateral load shifting while seated; (ii) load shifting while standing (latero-lateral and antero-posterior to simulate the balance control performed during the day); (iii) load control during sit-to-stand; (iv) gait swing and loading phase response (to stimulate a correct load shifting during the swing and stance phase); and (v) latero-lateral load shifting with knee flexion.

### 2.2. Usual Care Protocol

The usual care training was planned to mimic the exercises and interventions performed in the exergaming biofeedback training. The training was based on exercises for balance control and aimed at enhancing ADL relying on parallel bars and both stable surfaces (i.e., steps) and unstable surfaces (i.e., oscillating platforms and various-sized fitballs), including the following exercises: (i) latero-lateral load shifting while seated (to stimulate core stability and control); (ii) load shifting while standing (latero-lateral and antero-posterior to simulate the balance control performed during ADL); (iii) load control during sit-to-stand; (iv) gait swing and loading phase response: to stimulate correct load shifting during the swing and stance phase; (v) latero-lateral load shifting with knee flexion; and (vi) balance control during stairs.

### 2.3. Assessment

The effects of treatment were assessed by a trained blinded physiotherapist (S.M.) with experience in neurorehabilitation and neurological examination. The assessment was performed immediately before treatment (t0), immediately after the ten sessions (t1–4 weeks), and at one-month follow-up (t2). The adherence was assessed with the Pittsburgh Rehabilitation Participation Scale (PRPS) at the end of each session, in both groups. We employed a comprehensive set of clinical scales to assess balance, clinical status, and ability in the activity of daily living:

- The Berg balance scale (BBS) is a measure consisting of 14 items that assess the ability of patients to maintain positions or perform movements of varying complexity, with a total score ranging from 0 to 56. The items are designed to reflect tasks from daily life or to test the ability to maintain a specific position for a set duration, thereby providing a comprehensive indication of balance and stability [20].
- The Canadian neurological scale (CNS) was developed to facilitate the evaluation and monitoring of the neurological status of stroke patients, assigning a score ranging from 1.5 to 11.5. This scale encompasses 10 clinical domains, including the level of consciousness, orientation, speech, and motor function of the face, arms, and legs, providing a comprehensive assessment of neurological deficits [21].
- The Barthel index (BI) measures functional independence and mobility across ten activities of daily living (ADLs), such as feeding, personal hygiene, dressing, sphincter control, toilet use, transfers, ambulation, and the ability to climb stairs. With scores ranging from 0 to 100, the BI quantifies the level of assistance required by the patient. It is widely adopted as a tool to assess functional disability in individuals undergoing rehabilitation following stroke and other neuromuscular or musculoskeletal conditions [22].
- The Rivermead mobility index (RMI), with its 15-item questionnaire format, is dedicated to quantifying motor disability in post-stroke patients, assigning scores from 0 to 15. It evaluates functional abilities such as ambulation, balance, and transfers, providing an overview of the patient’s mobility [23].
- The National Institutes of Health Stroke scale (NIHSS) evaluates neurological deficits and recovery in stroke patients through its 15 elements, assigning a score from 0 to 42. It covers various areas such as level of consciousness, eye movements, visual fields, facial motility, limb strength, sensation, ataxia, linguistic abilities, speech, and spatial attention. Originally conceived to measure outcomes in clinical trials, the scale is increasingly used in clinical practice for initial assessment and planning of post-acute care [24].

#### 2.3.1. Postural Assessment

For postural assessment, we used a computerized platform equipped with a force plate that incorporates piezoelectric transducers. These can record the vertical component of the forces exerted on the platform surface, thereby enabling the detection of CoP at the contact area level. This device is connected to a computer equipped with dedicated software that analyses the movement of the patient’s center of mass and their oscillations during the recording period. Using this technology, CoP analyses were conducted, including the measurement of path length in millimetres (CoP_length_). The test, lasting 51.2 s, was performed barefoot, with three sessions, with both open eyes (OE) and closed eyes (CE), to calculate the average of the obtained values. During the tests, participants had no access to supports or aids, and the test was conducted in an isolated and quiet room, with the aim of avoiding any disturbances or distractions that could negatively impact their postural stability [25].

#### 2.3.2. Participation Assessment

The Pittsburgh rehabilitation participation scale (PRPS), through therapist observation, evaluates the level of patient participation in therapeutic activities. Based on a six-point scale ranging from “Poor” to “Excellent,” this scale measures the patient’s effort and engagement in the rehabilitation process [26]. Assessments using the PPS are conducted at the end of each therapy session.

### 2.4. Statistical Analysis

Distribution analysis was performed via the Shapiro–Wilk test, which showed a non-normal distribution in the general dataset. The rank transformation of baseline score and delta score (t1 − t0 and t2 − t0) did not show homogeneity in the regression slope, excluding the possibility to perform a non-parametric analysis of covariance (ANCOVA) using a pretest value like covariate [27].

According to the normality test results and the limited sample size, a non-parametric approach has been used. The Mann–Whitney U test was chosen to compare the average (μ) between the two groups (SB-group vs. UC-group) in the three evaluation times (t0, t1, and t2) as non-parametric between-group analysis. All results with *p* < 0.05 were considered statistically significant. The effect size was calculated using the *r* value comparing intra-group change between baseline (t0) and post-treatment (t1) and follow-up evaluation (t2) as follows:

$$r=\frac{Z}{\surd N}$$

where *Z* is the standardized test statistic value, and *N* is the total sample size. We interpreted the magnitude of the effect, such as: *r* = 0.1 to 0.3: small effect size; *r* = 0.3 to 0.5: medium effect size; *r* > 0.5: large effect size [28].

The average of PRPS score recorded in each session was compared between-groups using the Mann–Whitney U test. The statistical analyses were performed with Jamovi software v2.3 [29]. All data were reported with mean ± standard deviation.

## 3. Results

**Figure 2:**
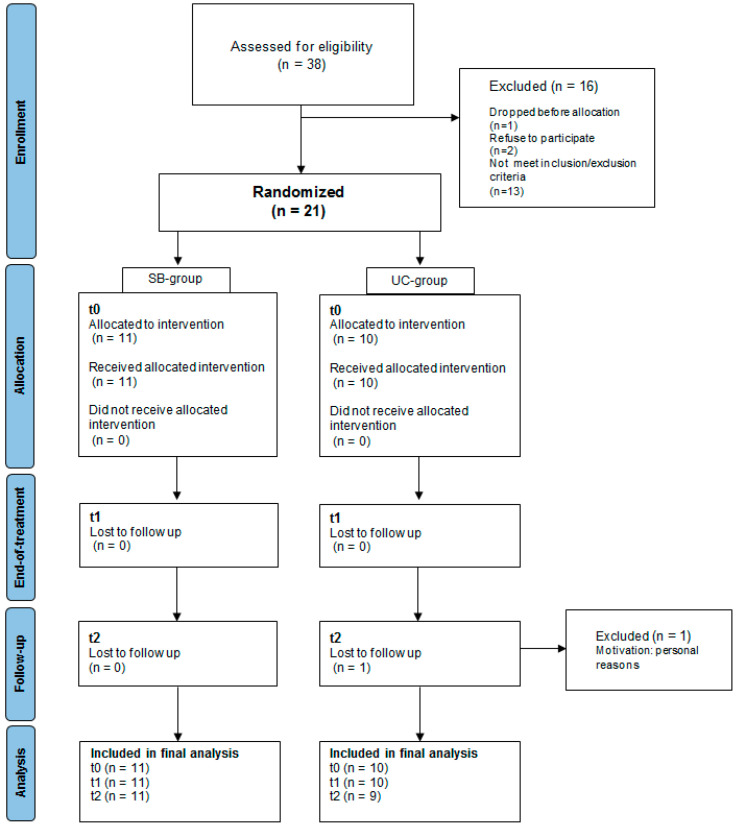
CONSORT diagram of patients’ allocation.

Thirty-eight patients were screened for the study. Of these, 13 did not meet at least one inclusion/exclusion criteria. Two patients refused to participate in the study for inability to reach the hospital. One patient dropped-out after their baseline evaluation, before allocation, for personal reasons. Finally, twenty-one patients (57.14 ± 13.82 years old) accepted to participate in the study and were randomly assigned into the two study groups. One patient in the UC-group refused the follow-up evaluation for personal reasons (for complete allocation see Figure 2). All patients provided a signed informed consent before to undergoing experimental procedures.

The patients were randomly assigned to two groups using a computer generated list. No differences in the demographic characteristics (all data are reported in Table 2) or clinical data were recorded at the baseline evaluation. All patients completed the 10 sessions of training without any adverse events.

### 3.1. Clinical Results

An improvement was observed in all clinical scales in both post-training and follow-up. However, no substantial differences were observed between the two groups, suggesting an overlapping in the gains across time (all results are reported in Table 3).

### 3.2. Postural Results

**Figure 3:**
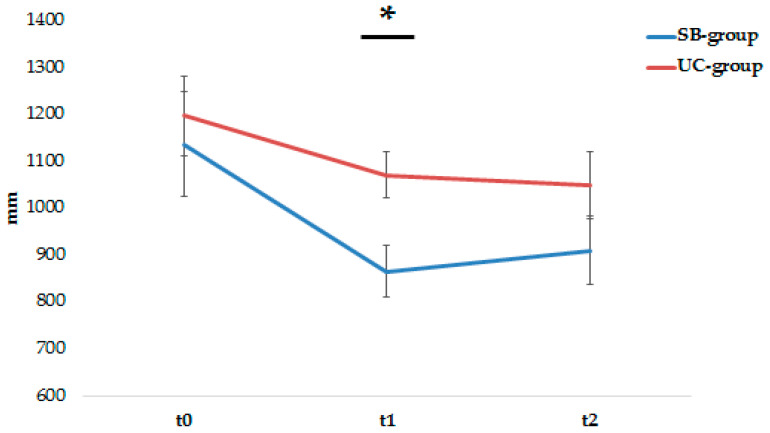
Changed in the length of CoP (expressed in mm) in the sensor-based group (SB-group) and usual-care group (UC-group) before (t0), after (t1) treatment and at one-month follow-up (t2). * *p* < 0.05.

In the postural assessment, statistically significant differences (U = 23; *p* = 0.02) were observed in the CoP_length_ with open eyes in the SB-group with respect to the UC-group. Specifically, a reduction in the CoP_length_ (SB-group: −270 mm; UC-group: −125 mm) was observed in the post-treatment evaluation (t1) with a large effect size (*r* = 0.69) (Figure 3). This gain between-group was maintained in the follow-up without statistically significant differences (U = 28; *p* = 0.11) and in the CoP_length_ with closed eyes (U = 33; *p* = 0.12).

### 3.3. Participation Results

Participation assessed with the PRPS showed a higher score in the SB-group (5.82 ± 0.27) with respect to the UC-group (4.92 ± 0.85), with a statistically significant difference (U = 14.5; *p* = 0.004).

## 4. Discussion

This study investigated the effects of sensor-based balance training with respect to the same dose-intensity of usual balance training in patients with chronic stroke. Both groups showed an improvement in both clinical and instrumental evaluations. No substantial difference was observed in the clinical improvement in the two groups. Differently, a statistically significant improvement in postural balance was recorded via a stabilometric platform in the SB-group. Finally, concerning participation level, in the SB-group a greater score was observed, indicating a higher compliance with the rehabilitation setting.

Postural analysis highlights that in chronic stroke patients, the sensor-based balance training can lead to a significant improvement in postural stability in the upright position, resulting in reduced body sway in the absence of movement [30]. During the quite upright position, the COP is considered to reflect partially the motor mechanisms that ensure balance, precisely the maintenance of the projections of the center of mass inside the feet base [31]. In this way, the CoP_length_ reduction can be interpreted as an improvement in dynamic postural balance, supporting the effectiveness of sensor-based balance training. Probably, the use of precise kinematic feedback can, via exergaming, intrinsically promote the recovery of postural balance during stance. In the condition with closed eyes, the difference did not archive the statistical significance in the two groups. This divergency can be explained by the predominant weight of visual afferents with respect to proprioception during upright position with open eyes [32]. In fact, when the upright position is maintained blindfolded, the proprioceptive system is less susceptible to external perturbation [33]. Regarding our result, the effect was observed in the open-eye condition since the proprioceptive system has been compromised by the predominance of visual afferent.

However, despite the difference in the result of postural stability, no other statistically significant differences were observed in clinical score between the two groups. Considering exclusively the clinical changes, the two groups improved after balance training, showing the same recovery pattern. This might suggest the efficacy of both approaches without one being more effective compared to the other one.

Our results were less pronounced and nearly comparable to the gains achieved in the control group with respect to the results of the same sensor-balance training performed on the sub-acute phase [34] of stroke. This discrepancy in the results obtained between the two disease phases could be attributed to the different physiological contexts characterizing the subacute versus the chronic phase of recovery. During the subacute phase, the higher permeability to rehabilitation is due to more significant cortical reorganization processes and more pronounced plasticity phenomena, making therapy via sensor-based balance training potentially more effective compared to the more chronic stages of the disease, as well as any rehabilitative approach [35,36].

Regarding participation level, the SB-group demonstrated a higher motivation during therapies, as evaluated by PRPS, when compared in the UC-group. The use of sensor-based training appears to enhance the level of motivation more effectively than conventional approaches. The increased effectiveness in terms of motivation can be attributed to certain characteristics of exergaming. Indeed, exergaming incorporates elements aimed at stimulating active participation of subjects through (i) differentiated difficulty levels, (ii) a playful atmosphere that encourages enjoyment, (iii) well-defined primary and secondary goals, (iv) a scoring system, and (v) a competitive environment that stimulates comparison with past performances [37]. These aspects not only facilitate a sense of continuous progression but also promote constant engagement thanks to the immersive experience, which captures attention, reduces distractions, and maintains high activity intensity [38]. Another significant advantage is the ability to customize exercise sessions directly on the screen in collaboration with the therapist, allowing greater flexibility and adherence to the therapeutic program [39]. These aspects can play an important role in rehabilitation, especially in patients with low compliance with conventional rehabilitative settings.

The feedback provided to the patient during technology-assisted therapy is fundamental for guiding the patient’s motor behavior and makes the therapy much more complete by eliciting multiple cognitive functions involved in learning processes [40]. Since the technology is increasingly widespread, it will be necessary to pay ever greater attention to this therapeutic determinant with the aim of characterizing it, both during the construction phase of the devices and during the therapeutic prescription phase [41,42].

### 4.1. Limitation

The sample size and the data distribution of the study imposes a non-parametric between-group statistical approach. Despite there being no statistical differences observed in demographic characteristics between the two groups, a divergency in stroke side and onset may affect the results. Considering the abovementioned limitations, the inferential power and the current conclusion should be interpreted as preliminary findings to drive future scientific investigations.

### 4.2. Future Perspectives

The effects observed in the SB-group on postural stability using a stabilometric platform suggest that instrumental analyses, due to their sensitivity in detecting changes, could be useful for more accurately quantifying the effects with respect to clinical ones. Therefore, future research should focus on the assessment of balance and postural stability functions using cutting-edge technologies (i.e., kinematics analysis) in clinical laboratory-like environmental settings [43].

## 5. Conclusions

In the present study, the effects of sensor-based balance training with respect to conventional balance training have been preliminary investigated in patients with chronic stroke. We observed a difference in postural stability in post-treatment evaluation in the SB-group with respect to the controls, which exhibited a less improvement. However, this difference was not observed at the follow-up. In the other clinical scales, no statistically significant differences were recorded. The results do not permit a judgment for the clear superiority of one approach over the other one. Regarding participation, the patients who underwent the sensor-based training showed a higher engagement, as shown by the higher score of PRPS with respect to the control group. These results suggest that sensor-based training can be a valid alternative to the traditional balance training where compliance might be compromised or reduced.

## Figures and Tables

**Table 1:** Eligibility criteria for participants.

**Inclusion Criteria**
Single event of cortical/subcortical ischemic stroke. Chronic phase of stroke (onset > 180 days) [17]. Lesion confirmed thought MR or CT. Able to stand upright with supervision or minimal assistance. Age between 18 and 80 years.
**Exclusion Criteria**
Severe general impairment or concomitant diseases (i.e., Parkinson disease). Orthopedic contraindications. Cognitive impairment (MMSE < 23) [18]. Diagnosis of unilateral spatial neglect.

**Table 2:** Demographics characteristics and group allocations.

ID	Gender	Age	Group	Stroke Lesion	Stroke Side	Onset (Months)	BBS	CNS	BI	RMI	NIHSS
3	F	51	SB-group	FTP	right	17	52	11	97	11	4
7	F	28	SB-group	LC	left	201	52	11	95	13	2
18	F	71	SB-group	FP	left	142	47	10.5	92	11	5
26	F	41	SB-group	IC-CR	right	70	45	8	98	9	1
27	M	71	SB-group	CN	right	10	23	6.5	66	6	8
29	M	57	SB-group	FTP-IC	right	6	16	6	48	4	9
31	M	65	SB-group	FTP	right	97	30	5.5	80	9	12
33	F	65	SB-group	FTP	right	48	34	7.5	79	10	8
35	M	45	SB-group	FP	right	6	36	8	69	7	9
36	F	47	SB-group	FTP	left	7	48	14	91	14	4
37	M	80	SB-group	IC-CR	left	6	35	6	64	7	5
		56.45 ± 15.56				55.45 ± 66.25	38 ± 11.93	8.54 ± 2.71	79.90 ± 16.43	9.18 ± 3.02	6.09 ± 3.36
5	F	37	UC-group	FTP	left	35	52	11	94	13	1
6	F	52	UC-group	FTP	right	13	50	10.5	94	13	1
12	M	60	UC-group	FTP	left	50	55	11.5	90	10	3
17	F	43	UC-group	FP	left	37	37	12	88	6	2
11	M	52	UC-group	CN	right	7	51	13.5	95	13	3
8	F	78	UC-group	CN	left	6	52	12.5	95	11	4
28	F	67	UC-group	FTP	left	7	25	5.5	61	6	11
30	M	67	UC-group	FTP	left	28	21	3	64	7	14
32	M	67	UC-group	FP	left	64	52	8	93	12	3
34	M	56	UC-group	FTP-CN	right	32	11	6.5	40	2	11
		58.11 ± 13.16				27.9 ± 19.77	40.6 ± 16.02	9.40 ± 3.46	81.40 ± 19.35	9.30 ± 3.83	5.30 ± 4.78
	*p* = 0.84	*p* = 0.81			*p* = 0.13	*p* = 0.22	*p* = 0.38	*p* = 0.44	*p* = 1	*p =* 0.86	0.39

Abbreviations: LC, lenticular capsule; FTP, fronto-temporo-parietal cortex; FP, fronto-parietal cortex; IC, internal capsule; CR, corona radiata; CN, capsular nucleus.

**Table 3:** Clinical and instrumented results.

Scale (Time)	SB-Group	Effect-Size (*r*)	UC-Group	Effect-Size (*r*)	*p* Value
BBS (t0)	38 ± 11.93		40.6 ± 16.02		0.376
BBS (t1)	42.18 ± 11.88	0.23	43 ± 14.51	0.23	0.697
BBS (t2)	41.63 ± 12.44	0.21	45.77 ± 12.65	0.40	0.619
CNS (t0)	8.54 ± 2.71		9.40 ± 3.46		0.437
CNS (t1)	9.09 ± 3.21	0.09	9.85 ± 3.83	0.14	0.672
CNS (t2)	9.13 ± 3.29	0.10	10.11 ± 3.80	0.17	0.621
BI (t0)	79.90 ± 16.43		81.40 ± 19.35		1000
BI (t1)	82.54 ± 17.45	0.18	83.70 ± 20.10	0.31	1000
BI (t2)	82.81 ± 17.42	0.20	88.11 ± 14.89	0.43	0.761
RMI (t0)	9.18 ± 3.02		9.30 ± 3.83		0.859
RMI (t1)	10.81 ± 3.40	0.32	10.20 ± 4.10	0.23	0.831
RMI (t2)	11 ± 3.22	0.36	11.44 ± 3.35	0.41	0.671
NIHSS (t0)	6.09 ± 3.36		5.30 ± 4.78		0.395
NIHSS (t1)	5.18 ± 4.02	0.17	4.30 ± 4.98	0.29	0.595
NIHSS (t2)	5.18 ± 4.02	0.17	3.55 ± 4.66	0.39	0.358
CoP_length_ OE (t0)	1137.34 ± 284.43		1197.30 ± 354.38		0.765
CoP_length_ OE (t1)	865.99 ± 160.81 *	0.69	1071.84 ± 174.73 *	0.32	0.024 *
CoP_length_ OE (t2)	911.40 ± 239.63	0.59	1050.17 ± 232.30	0.29	0.112
CoP_length_ CE (t0)	1361.55± 366.87		1505.53 ± 606.02		0.512
CoP_length_ CE (t1)	1110.65 ± 199.54	0.37	1309.75 ± 313.56	0.20	0.132
CoP_length_ CE (t2)	1020.67 ± 234.82	0.57	1140 ± 218.37	0.49	0.261
PRPS	5.82 ± 0.27 *		4.92 ± 0.85 *		0.004 *

Abbreviations: BBS, Berg balance scale; CNS, Canadian neurological scale; BI, Barthel index; RMI, Rivermead mobility index; NIHSS, National Institute of Health Stroke Scale; CoP_length_, length of center of pressure; OE, open-eyes; CE, closed-eyes; t0, baseline evaluation; t1, post-treatment evaluation; t2, follow-up evaluation. * *p* value < 0.05 (this result refers to the non-parametric between-groups comparison).

## Data Availability

The raw data supporting the conclusions of this article will be made available by the authors on request. The data are not publicly available due to local privacy policy.
